# Meta-Analysis of the Clinical Effect of MIS-TLF Surgery in the Treatment of Minimally Invasive Surgery of the Orthopaedic Spine

**DOI:** 10.1155/2022/2315533

**Published:** 2022-03-16

**Authors:** Wanliang Yang, Xin Pan, Xun Xiao

**Affiliations:** ^1^College of Clinical Medicine, Shandong University, Jinan 250000, Shandong, China; ^2^Qilu Medical College of Shandong University, Jinan 250000, Shandong, China

## Abstract

Minimally invasive surgery (MIS) has already had a significant impact on surgical treatment (spine). Because they are less invasive, minimally invasive treatments are often preferred over open spine surgery. MIS and open spine surgery in terms of posterior lumbar fusion (PLF), lumbar disc herniation (LDH), and cervical disc herniation (CDH) were all observational studies based on randomized controlled trials. Seventeen RCTs and six observational studies were conducted. Chemotherapy had no effect on the long-term alleviation of the neck or arm pain in patients with CDH. In LDH, MIS was superior in terms of pain relief, rehospitalization rates, and improvement in quality of life. At the expense of increased perioperative endoscopic, readmission, and revision rates, MIS achieved a significant reduction in 2-year expenditures, fewer medical problems, and improved Oswestry score ratings. There is no evidence to support the use of MIS over open surgery for lumbar or cervical process disc herniation. In comparison, MIS-TLIF has several advantages, in addition to lower revision/readmission rates. However, MIS significantly increases the surgeon's radiation exposure, regardless of the patient's sign. However, the effect on patients is unknown. These findings could help patients make better decisions when comparing open spine surgery to minimally invasive spine surgery, especially given how much advertising is out there for MIS.

## 1. Introduction

Historically, “open surgery” has been the method of choice for spine surgery. A lengthy incision must be made in the area to be operated on for the surgeon to be able to examine and analyze the anatomical structures. More back and neck disorders may now be treated with minimally invasive surgery thanks to recent technical advances. Because MISS does not require a long incision, it reduces the risk of substantial damage to the muscles around the spine. Pain and recovery time are reduced. The indications for minimally invasive spine surgery are similar to those for open surgery. Spine surgery is usually shown only after nonsurgical treatments like drugs and physical therapy have failed to relieve severe symptoms. Also, surgery is only shown if your doctor has discovered the specific cause of your pain, such as a herniated disc or spinal stenosis. There are a variety of minimally invasive techniques available. Every one of them has one thing in common: reduced muscle injury and smaller incision size. Using minimally invasive procedures to undertake common operations such as lumbar decompression and spinal fusion, which are both effective, decompression lowers pressure on the spinal nerves by removing bone fragments or a herniated disc. This procedure is used to correct problems with the spine's small bones (vertebrae). The goal is to heal the painful vertebrae and then fuse them together to form a single strong bone structure. The goal of this article is to explore spinal decompression and fusion techniques that are minimally invasive. MISS (minimally invasive spine surgery) is another term for this procedure. Using specialized tools, surgeons can reach the spine through small incisions during these operations. During open surgery, the doctor makes a six-inch incision or less and then moves the muscles to the side to see the spine. The surgeon can now reach the spine and remove sick or injured bone or intervertebral discs using the side muscles. Additionally, the surgeon can see right away where cages, bone grafts, and screws are needed to supply support and ease healing of the spinal bones throughout the procedure. Open surgery may cause a significant level of criticality because of the possibility of the muscle being harmed during the tugging or “retraction” procedure. While muscle retraction is designed to aid the surgeon in finding the location of the problem, it often affects anatomy more than the surgeon needs. Therefore, muscle injury is more likely to occur because of this, and patients may feel postoperative pain that is distinct from the back discomfort they were experiencing before the surgery. It is possible that this will result in an extended recovery time. Added complications may include higher blood loss and infection risk because of the larger incision and harm to soft tissues. This technique was created to treat spinal illnesses while minimizing injury to muscles and other natural spine systems. Additionally, it allows the surgeon to see only the part of the spine that is affected by the problem (the problem area). In addition, MIS offers the advantage of requiring fewer incisions, resulting in less bleeding and requiring fewer hospital stays. The most common technique is the tubular retractor. A tiny incision is made and a tubular retractor is inserted into the spinal column through the skin and soft tissues. This builds a tunnel to the affected area of the spine. The tubular retractor keeps the muscles relaxed throughout the process. The surgeon gets access to the spine using small devices that are integrated into the tubular retractor's central core. To install fusion devices such as screws or rods, the retractor must first be removed from the area of the injured bone or disc. For some surgeries, many retractors or incisions may be needed.

The surgeon uses fluoroscopy to guide him through the process of creating the incision and inserting the retractor. During the surgery, a screen shows the surgeon real-time X-ray pictures of the patient's spine. During surgery, the surgeon will typically use a microscope to examine the spine's key components, such as the discs and joints. After surgery, the tubular retractor is withdrawn and the muscles are allowed to heal. The likelihood of muscular injury is more common with open surgery; in the spine surgery sector, new minimally invasive procedures are constantly being developed. An endoscope, for example, is currently being used by some spine surgeons to gain access to a problem site in the spine. Anesthesia for MISS is most administered as either general anesthesia (during which you stay unconscious) or regional anesthesia (during which you are awake during the procedure).

### 1.1. Overview of the Manuscript

In this manuscript, the meta-analysis of the clinical effect of MIS-TLIF surgery in the treatment of minimally invasive surgery of the orthopaedic spine and the common comparison between the MIS and the TFIL are discussed in the following section, which is followed by the literature survey. Participation in the research is subject to certain requirements. Extraction of information, the quality of the research, and analytical statistics are used to analyze data. Evidence levels are used to categorize the effects of research investigations, and patients and the process of choosing the method are discussed as follows.

## 2. Minimally Invasive Spine Surgery Is a Common Surgery

The compression of a nerve by a herniated disc in the lower back can cause severe leg discomfort, numbness, and even paralysis in certain cases. To ease these symptoms, the disc is surgically removed. A diskectomy is the medical term for this procedure.

To remove the herniated disc, the patient is turned face down and a tiny incision is made. During the surgery, a small part of the lamina bone is removed. This gives a better view and an idea for the surgeon to visualize the spinal nerve and the disc. A surgeon (doctor) delicately retracts the nerve and removes only the affected disc, leaving the rest of the nerve intact. It is also possible to employ this minimally invasive method to repair herniated cervical discs in the neck. The technique is known as MIS posterior cervical foraminotomy/discectomy.

Open lumbar fusions may be done via the back, belly button, or side. Lumbar fusions may also be performed minimally invasively. The surgeon can approach the spine from the side, reducing the amount of spinal nerve displaced during the surgery enabled by the transforaminal lumbar interbody fusion (TLIF). After the patient is placed face down during a MIS-TLIF, the surgeon installs one retractor on each side of the spine, completing the procedure. This avoids rupturing the midline ligaments and bone. The surgeon removes the lamina and disc, then places the bone transplant into the disc region, fastening it with screws or rods as needed. To boost the likelihood of healing, the surgeon may choose to use a bone transplant in addition to the patient's own bone on occasion. Aside from being performed minimally invasively from the side, spinal fusions are also performed regularly. The side approach is enabled by the Direct Lateral Interbody Fusion (DLIF) and Extreme Lateral Interbody Fusion (XLIF). They are superior to conventional spinal fusion procedures in that they do not injure the back muscles or strain or pull on the spinal canal nerves.

An oblique lateral interbody fusion is a more contemporary variation of this surgery that is performed on the left side of the body (OLIF). OLIF, like XLIF and DLIF, needs the use of a side incision. Instead, OLIF enters an oblique position, avoiding the psoas muscle (the muscle on the side of the spine). All three of these lateral strategies produce outcomes that are comparable. MISS, like any other operation, entails some inherent hazards that must be understood and accepted. Some studies show that MISS has similar issues to those associated with open spinal fusion surgery, but some studies show that MISS has a lower infection rate than open spinal fusion surgery. Before surgery, your doctor will go over each risk with you in detail, and they will take specific precautions to help avoid any negative outcomes.

## 3. The Following Issues May Arise Because of MISS

Antibiotics are given to the patient before, during, and often after the surgery to lower the risk of developing an infection. It is normal to experience some bleeding after surgery, but it is usually not significant. There is a pain in the area where the graft was placed. Occasionally, a small number of people have persistent discomfort at the location of their bone transplant. Some symptoms can recur on a regular basis. Some people may revert to their first symptoms. Pseudarthrosis, insufficient bone growth, causes incomplete spinal fusion healing. If this happens, further surgery may be necessary to produce a solid bone union. Pseudarthrosis is more frequent among smokers, who are more susceptible. Nerve injury is a big issue. These operations may injure nerves or blood vessels. These kinds of ramifications are quite uncommon. Blood clots are formed. It is also possible that blood clots will form in the legs because of surgery, which is unusual. If they shatter and travel to the lungs, they are a major hazard to the patient's health. Minimally invasive procedures can result in shorter hospital stays since they are less intrusive. MISS patients typically return home on the same day or within one to two days of being admitted to the hospital. The exact length of their hospital stay varies depending on their condition and therapy. Most patients who undergo conventional surgery are admitted to the hospital for three to five days after the procedure.

Because minimally invasive therapies do not damage muscles or soft tissues, postoperative discomfort is believed to be less severe than that experienced following traditional open procedures in most cases. While you can expect some discomfort, current advances in pain management help your doctor manage and treat your pain. Physical therapy may be recommended by your doctor to aid you in regaining strength and speeding up your rehabilitation. Depending on the procedure and your overall health, this will vary. Specific workouts might aid you in regaining the strength you need to return to work and your normal activities after an injury. It might take months for the bone to harden after a fusion. On the other hand, your degree of comfort will often improve far more quickly. For the fused spine to heal properly, it is vital that it keeps proper alignment throughout the healing process. On the job, you will learn to move appropriately, reposition, sit, stand, and walk. The amount of time it will take you to return to your typical activities after a MISS varies based on your unique operation and condition. In the days after surgery, your doctor will check on you to ensure you are recovering well.

Medical and surgical therapies are evaluated objectively through research. The quality of evidence decides how much research impacts management. Class I data from prospective randomized controlled trials (RCTs) are the most persuasive in showing a treatment's effectiveness. In this research, the following conditions are analyzed, such as [1]. Five studies comparing MIS with open surgery for CDH were chosen as Class I trials. Four RCTs and systematic analysis were included. The RCTs included 200+ MIS patients and 150+ open surgery patients, following them for more than 100 weeks (about 2 years) [2]. Only one of the four RCTs (8 percent) used a discectomy without fusion on 19 of 200+ open surgery participants. The remaining 150+ patients had anterior cervical discectomy. Only 37 out of 200+ MIS patients (15%) had fusion, while 100 out of 150+ had posterior foraminotomy. A posterior cervical discectomy was performed on 60 individuals, and an amniotomy or discectomy was performed on 18 patients (specific procedure per patient not reported). In RCTs, only 95 of 200+ MIS patients (42%) employed an anterior approach, compared to 190 of 200+ conventional open patients (88%) [3, 4].

This review shows that MIS did not enhance neck pain and arm discomfort, compared to TLIF for CDH. While MIS helped relieve temporary neck discomfort, when lumbar patients were included in the pooled estimate analysis, this improvement was not statistically significant. Minimally invasive vs. open surgery Twelve trials comparing MIS with treatment of LDH produced Class I evidence. It includes ten RCTs and two systematic reviews. In the 10 RCTs, 489 individuals receiving MIS and 500+ patients with traditional open surgery were recruited in the 10 RCTs. Both groups did not obtain fusion. Out of 500+ open patients, 500+ got discectomy, and 19 had percutaneous nucleosome, with the follow-up durations of 50–100 weeks (about 2 years) [4–6].

On average, MIS is inferior to open LDH surgery for leg discomfort, less back pain, lifestyle and quality of life, and rehospitalization. Conversely, MIS was linked to a decreased risk of infection and a reduced hospitalized duration. Postoperative Oswestry Disability Index (ODI) scores were not altered. The authors suggest that any alleged decreased infection incidence is linked with MIS. When compared to open surgery, MIS exposed the surgeon's thyroid and eye more than tenfold, his chest 14-fold, and his hand 20-twofold. Surgery with a minimum of invasiveness against surgery with a maximum of invasiveness: disc herniation is a condition in which the disc protrudes from its normal location (cervical or lumbar) [5, 6]. Data from 14 RCTs on CDH and LDH were combined to form a single comprehensive assessment. When compared to traditional open surgery, the study discovered that minimally invasive surgery (MIS) decreased infection rates while increasing nerve root damage, durotomy, and reoperation rates. However, none of these changes were statistically significant. At the rear of the neck, the lumbar spine is fused [7].

## 4. Literature Survey

Lumbar fusion surgery treats spinal instability, stenosis, spondylolisthesis, and degenerative disc disease symptoms, normalizing motion and stability while keeping load-bearing ability and aligning the system. Lumbar fusion operations have grown in popularity in the US during the last several decades [8].

Transitioning from traditional open surgery to less invasive approaches is the method of choice for many surgical procedures. According to postoperative histology and imaging examinations, standard open procedures leave traces of scar during tissue development, muscle retraction, and stripping, all of which have a negative impact on outcomes and increase the likelihood of reoperation. To help maintain paraspinal muscular anatomy and bone architecture, minimally invasive treatments employ a muscle-dilating approach. These treatments have been proven to reduce the incidence of iatrogenic soft-tissue injuries [9] [6–8]. Reduced postoperative discomfort, decreased intraoperative blood loss, a shorter postoperative staying in hospital, fast recovery to normal activities, and a lower reoperation rate are just a few of the reasons that minimally invasive spine (MIS) therapies are becoming increasingly popular. Due to the lack of long-term data on patients receiving minimally invasive spinal fusion for severe back pain, the use of these procedures instead of standard open fusion methods continues to be controversial in the medical community. We want to add to the data in this discussion by supplying long-term, prospectively recorded results from one of the biggest presently known series of MIS-TLIF by following patients up to 28 months (about 2 and a half years) [10]. According to our findings, MIS-TLIF beat open TLIF in all domains, except for the fusion rate. As reported in the meta-analysis, the MIS-TLIF procedure had an identical fusion rate as the open TLIF procedure but with a shorter hospital stay, faster ambulation, and less blood loss [11]. Staying in the hospital for less time also reduces medical expenditures because MIS-TLIF enhances ambulation speed [12]. The MIS group's postoperative VAS and ODI scores were lower than the open groups. Although the preoperative clinical and functional baseline measures were equal across the groups, the open group's postoperative ratings were lower than those of the MIS groups. So, MIS-TLIF improved outcomes while reducing trauma [13].

In recent years, TLIF has gained widespread acceptance and recognition as a surgical method that significantly reduces the likelihood of relative nervous system disorders, despite the advancement of fusion technology. However, the open TLIF technique requires paraspinal muscle splitting to be successful. It does not hurt, but it ruptures a large piece of the posterior compartment, causing muscle stiffness and low back discomfort. As a result of its advantages over standard open surgery, such as less harm to spinal soft tissues and paravertebral muscles, it has gained popularity in recent years. The advantages of MIS-TLIF can be linked to the fact that less paravertebral muscle dissection and retraction occur throughout the procedure [14].

According to the study, it is found that the MIS-TLIF and open TLIF have no difference in the surgery time, complication rates, or reoperation rates. Despite this, the compilation and reoperation rates for the MIS are better than the open TLIF process. One of the most persuasive arguments is that the learning curve for minimally invasive surgical abilities is steep, requiring added years of experience to perfect the necessary skills [15]. The advancement of surgical devices and technology may pave the way for realizing the other critical goals. Our meta-analysis has several flaws that need to be addressed. Because we only looked at prospective and retrospective research, issues including data bias, nonblinding or improper, inadequate baseline comparisons, and data collection must be addressed. For the second time, the current study relied on a small number of outcome indicators; clinical outcomes should be assessed by a variety of alternative aims or subjective characteristics. In addition, there were inconsistencies in the definition and assessment of fusion during the study. A further point to mention is that many of the investigations were completed in a short or medium amount of time. More long-term follow-up studies must be reviewed to analyze the evaluation process for the effectiveness of these measures. Finally, an inherent bias was added to the data by combining them all together [16]. Data collection and inappropriate or nonblinding were challenges that arose due to solely looking at prospective and retrospective studies. The use of MIS-TLIF is also not related to an increase in complications or reoperations. Comparing open surgery and MIS, there are fewer lesions, better outcomes, and the same fusion rate. Surprisingly, MIS-TLIF has been linked to fewer complications and reoperations. This may be due to increased acceptability of MIS-TLIF, familiarity with the surgery, ability in MIS abilities, and development of proper surgical instructions and instruments. Added high-quality research is needed to confirm and compare these findings [15, 16]. We searched the CNKI, Wanfang, and VIP databases for relevant information using the keywords “MIS” and “TLIF”. Many researchers recommend restricting the search to English-language articles. The retrieval period ran from the database's creation until January of the current year. It was also searched for articles related to the original research and review papers in the reference lists of those articles. Two reviewers independently reviewed all publications based on their titles, abstracts, or full texts. The two reviewers assigned to them knew the writers and their journals [17]. On a particular research project, diverse viewpoints were heatedly debated until an agreement was set up.

### 4.1. Participation in the Research Is Subject to Certain Requirements

The criteria for consideration were as follows: (3) at least one clinic result or perioperative data were presented in the article; (4) the patients had degenerative disc disorders (disc herniation, canal stenosis, or spondylolisthesis); this study excludes the patient who underwent open TLIF or MIS-TLIF for other illnesses. These patients were likewise eliminated from the study [18, 19].

### 4.2. Extraction of Information

From the research that was included, we were able to extract information for the categories listed as follows: (1) the names of the authors and the year of publication; (2) the study strategy; (3) the type of evidence; (4) the total number of patients recruited, as well as the number of patients in each group (MIS-TLIF versus open TLIF); (5) the average amount of time spent following up; (6) the average follow-up time and rate (in percentage points); (7) the patients' average age; (8) the proportion of patients who are male and female; (9) predictive diseases (like degenerative disc disease, spondylolisthesis, and others are classified into three categories); (10) clinical outcomes; (11) perioperative data points; (12) inclusion/exclusion criteria; (13) information about the postoperative period; (14) the total number of lumbar segments treated; (15) the definition of fusion and the assessment of the measure; (16) the types and quantity of grafts used; (17) the number and type of cages used; (18) the screw fixation technique and the number of issues met; (19) the number of complications met. We had not predicted any problems ahead of time. Moreover, the total number of challenges was tallied up for analysis [19, 20].

### 4.3. The Quality of the Research

Levels of Evidence (2009) Class I claims were supported by high-quality randomized controlled studies (RCTs). Class II evidence includes RCTs of moderate to inferior quality and correct cohort studies. Class III evidence includes cohort and case-control studies of moderate or inferior quality. The Case Series Study was in Class IV Evidence. Two reviewers separately evaluated the publications, and disagreements were discussed until a consensus was reached [21, 22].

### 4.4. Analytical Statistics Are a Type of Statistics That Is Used to Analyze Data

WMDs were used to measure the ODI, average blood loss, hospitalized duration, time taken for the surgery, and VAS. The relative risk (RR) measure and 95% confidence interval were used for dichotomous data. Each study has its own unique set of criteria for evaluating the efficacy of the fusion procedure. Consequently, the total number of studies within each category was not equal. In this research, a random-effects model was preferred over a fixed-effects model because it supports a distribution that the fixed-effects model does not. All tests required a *p*-value of 0.05 or less. The publication bias was investigated using funnel plots. Asymmetry suggests a publication's prejudice, while symmetry implies no bias. We could conduct added database analysis using Review Manager [5].

### 4.5. Evidence Levels Are Used to Categorize the Effects of Research Investigations

Randomized double-blind placebo-controlled trials are appointed as Class 1 trials (randomized outcome measures) [23]. Experiments were conducted without a randomization procedure (Class II). Unlike Class I, this group is not random. Case-control, cohort, and interrupted time studies processed with controls are all examples of Class III Observational Studies. Observational studies without controls are Class IV (Class III, without controls) and Ability (Class V) (invited only can comment).

## 5. Patients and the Process of Choosing the Method

Between 2003 and 2010, 318 MITLIF surgeries, as shown in Table 1, were conducted on 304 consecutive patients, all of whom were treated using a paramedian muscle-sparing technique. During the operation, 120 men and 184 women were included in the research. The mean age (range, 19–93) and gender (male) at surgery were 62.4 and 19.4, respectively. The senior author performed all 318 MITLIF surgeries on all 318 patients from an outpatient neurosurgery spine clinic. Following a thorough clinical history, physical exam, and lumbar spine radiological study, the diagnosis was made. When spondylolisthesis and retrolisthesis were diagnosed, they were classified according to the Meyerding classification (I–IV). All the patients were examined outside of a hospital environment.

Complicating matters, the patient also has several medical conditions, which are listed in the table. Spondylolisthesis (66%) was the most common clinical condition, followed by central spinal stenosis (47%) and foraminal stenosis (34%). The most common clinical findings were persistent severe low back pain, neurogenic claudication, and radiculopathy-related symptoms [5]. Patients experienced symptoms for an average of 68.6 months (about 5 and a half years) before surgery. Previously, 70 individuals had lumbar surgical operations performed on them (23%). When clinical and imaging data agreed, as well as symptoms that had lasted for more than six months and had not responded to nonoperative treatment, patients were considered candidates. The table shows patients who had conservative nonoperative therapy. If a neurological impairment was deteriorating or if the patient presented with considerable, incapacitating pain and a good correlation between clinical and radiographic data, surgical therapy was started no sooner than 6 months. If nonoperative treatment fails, surgical intervention is recommended as the last resort for patients who were physically unsuited for surgery, patients who had a recent bleeding diathesis, patients who were infected, patients who had incongruent clinical and radiographic data, and patients who were infected [5, 22, 23].

In the field of outcome measurement, outcome measures are a type of outcome measurement. Low back pain, back-related functional disability, and physical and mental quality of life were all improved when the visual analogue scale (VAS), the ODI, and the Short-Form 30+ were used to assess patients' reported results (SF-36). During the recruitment process, patients were requested to complete these validated questionnaires, and they were also asked to complete them again at various stages throughout the postoperative period. The presurgery scores, all later follow-up, and change scores were all analyzed for significance. A secondary outcome measure was the rate of fusion, the rate of reoperation, the amount of intraoperative blood loss, and the length of time spent in the hospital following the procedure. To assess fusion, radiologists separately and blindly reviewed radiological images taken from the lumbar anterior-posterior and lateral flexion/extension views during the postoperative phase. We considered fusion effective when there was no considerable motion or angulation at the fused level, no implant latency, no hardware loosening or breakage, and bridging bone growth.

## 6. Data Analysis Using Statistical Methods: Analyzing the Information

To compare the ODI, SF-36, and VAS values for each time, Student's *t*-test was used. Consideration is decided based on the *p*-values, i.e., *p*-values <0.05 are noted as significant and *p*-values >0.05 are noted as highly significant.

On the other hand, three RCTs compared MIS-TLIF and open TLIF. Both open surgery and MIS were inspected for an average of 25 months (about 2 years). On postoperative day 2, blood loss and back pain were much lower in individuals receiving minimally invasive surgery (MIS), although it took significantly longer to supply intraoperative radiotherapy. Because all patients underwent open lumbar spine surgery prior to being randomly distributed to a group, the research had significant limitations. There was no difference in operating time, blood loss, or postoperative hospitalization between minimally invasive and open surgery. MIS needed less postoperative drainage and recovery time (40 days (about 1 and a half months) vs. 76 days (about 2 and a half months), but more time for intraoperative fluoroscopy. The VAS evaluations did not vary between the two groups at 3, 6, 12, and 24 months (about 2 years) postoperatively, but the ODI did. Four years after surgery, a study of 41 patients (21 with MIS and 20 with open TLIF) found no clinically significant differences between the open TLIF and MIS groups. In terms of narcotic independence, two-year social cost, and return to work, numerous nonrandomized prospective studies have set up that MIS-TLIF is better. MIS was comparable to open TLIF in terms of operating time, complication rate, and reoperation rate but with reduced blood loss and reduced hospital stay. In the second meta-analysis of 20+ trials, the surgical team received higher radiation. In a recent PLIF trial, it was found that MIS and open fusions have no differences. MIS had a higher revision and readmission rates than those in open TLIF and PLIF.

## 7. Conclusion

The strongest evidence does not confirm that the MIS process is better than open surgery for cervical or lumbar disc herniation. On the other hand, increased revision, readmission rates, reduced hospitalization, cost of treatment, and time to reach the normal life are disadvantages of MIS-TLIF fusion. No RCT comparing MIS-TLIF only to open PLIF has been conducted, which would supply valuable information. Any surgery involving MIS exposes the surgeon to much higher radiation. This is especially true for lumbar spine surgery, which is quite common. Whether this exposure had any effect on the patients is unknown at this time, and more research is needed. Patients must be informed of the most recent results to choose between MIS and open spine surgery or TLIF. This is critical given the current medical advertising climate that heavily promotes MIS.

## Figures and Tables

**Table 1:** Recommendation for patient population.

Recommendation	Population of the patient
As compared to open surgery conducted normally, MIS	Cervical disc herniation
1. It has no effect on short-term functional outcomes.	
2. It does not have a negative impact on long-term performance.	
3. It does not alleviate acute arm discomfort.	
4. It does not supply relief from persistent arm discomfort.	
5. It supplies immediate relief from severe neck aches.	
6. Does not supply relief from chronic neck pain.	
When compared to conventional open surgery, MIS	Lumbar disc herniation
1. It does not improve function in the short term.	
2. It does not have a negative impact on long-term performance.	
3. It does not supply enough relief for leg pain.	
4. Low back pain is not well relieved by this medication.	
5. It has a greater likelihood of requiring rehospitalization.	
6. The quality of life is negatively affected by the procedure.	
7. The chance of surgical site and infection issues is reduced.	
8. It is possible that it is associated with a shorter period of hospital stay.	
9. It does not show Oswestry Disability Index scores after at least six months after surgery interns of statistically significant difference.	
10. In addition, it exposes the surgeon to more than ten times the amount of radiation that would otherwise be supplied to the thyroid or the eyes.	
11. Exposure to more than 14 times the quantity of radiation supplied to the chest is imposed on the surgeon.	
12. Subjects the surgeon's hand to more than 22 times the amount of radiation received by the patient.	
According to the existing practice of open surgery, as compared to open surgery as it is now practiced,	Disc herniation
1. The incidence of nerve root damage is growing.	
2. The incidence of nerve root injuries is decreasing.	
3. A rise in the number of inadvertent durotomies that occur	
4. Increased reoperation rates are becoming more common as a trend.	
5. Infection-prevention trends in the United States	
When comparing MIS with TLIF and open TLIF	Posterior lumbar fusion
1. leads to much less blood loss than the latter.	
2. It leads to a considerable reduction in back pain on the second postoperative day after surgery.	
3. It involves much greater amounts of intraoperative radiation time.	
4. It does not need a major increase in overall operating time.	
5. Despite considerable improvement in the ODI over a brief period, there is no statistically meaningful difference in terms of long-term clinical outcome.	
6. There is no statistically significant difference in the radiographic outcome.	
7. It has led to reducing the number of hospitalizations of the patients	
8. It has reduced the amount of time necessary to a normal life routine.	
9. It has reduced indirect expenses.	
10. During a two-year period, he has cut social expenses.	
11. It has resulted in increased narcotic independence	
When compared to open TLIF/PLIF, MIS-TLIF/PLIF has the following benefits over the former:	Posterior lumbar fusion
1. A faster rate of revision.	
2. A higher risk of hospital readmission.	
3. There has been no change in the incidence of surgical complications since the study began.	
4. A reduction in the number of medical problems	

The most persuasive evidence supporting MIS compared to open spine surgery in the patient group suffering from CDH, LDH, and PLF is reviewed.

## Data Availability

The data that support the findings of this study are available from the corresponding author upon reasonable request.
